# New insights into the putative XX/XY sex chromosomal system in blue‐eyed red‐fin pleco *Hypostomus soniae* (Siluriformes, Loricariidae)

**DOI:** 10.1111/jfb.70226

**Published:** 2025-09-13

**Authors:** Luan Aércio Melo Maciel, Renata Coelho Rodrigues Noronha, Bruno Rafael Ribeiro de Almeida, Manoella Gemaque Cavalcante, Cleusa Yoshiko Nagamachi, Julio Cesar Pieczarka, Luís Reginaldo R. Rodrigues

**Affiliations:** ^1^ Program in Society, Nature and Development Federal University of Western Pará Santarém Brazil; ^2^ Laboratory of Genetics and Cell Biology Center for Advanced Studies in Biodiversity, Federal University of Pará Belém Brazil; ^3^ State University of Pará, Campus XVIII Cametá Brazil; ^4^ Laboratory of Cytogenetics Center for Advanced Studies in Biodiversity, Federal University of Pará Belém Brazil; ^5^ Laboratory of Genetics and Biodiversity Institute of Educational Sciences, Federal University of Western Pará Santarém Brazil

**Keywords:** Cytogenetic, in situ hybridization, Loricariidae, recombinant DNA (rDNA), Synaptonemal complex, Tapajós River

## Abstract

Blue‐eyed red‐fin pleco *Hypostomus soniae* (family Loricariidae) presented a putative sex system XX/XY in early stage. Aiming to explore the inter‐populational karyotypic variation and proposed emergence of the XX/XY system, we studied 13 *H. soniae* individuals (6 males, 7 females) from the Tapajós River. Mitotic karyotypes and meiotic cells were analysed using C‐banding, site verification Ag‐NOR (nucleolar organizing region), chromomycin A_3_ (CMA3) and fluorescent in situ hybridization with repetitive DNA probes (DNAr 18s, 5S, histone H1, H3, telomere). The synaptonemal complex on meiocytes was studied using immunodetection with anti‐structural maintenance of chromosomes protein 3, anti‐γH2AX and anti‐H3K9ac. The karyotype presented was 2*n* = 64, with clear size heteromorphism varying between male and female. The species presented multiple NORs colocalized with 18S and CMA3‐positive marks in two pairs of acrocentric chromosomes. The centromeric region of pairs 25 and 26 carries the repeats of histones H1 and H3. All the bivalents at pachytene exhibited tip‐to‐tip pairing, revealing the absence of an XY pair with partial synapsis. The synaptic behaviour of the putative sexual pair failed to corroborate the XX/XY system hypothesis in *H. soniae*. Further cytogenetic and molecular investigations are necessary to determine the proposed emergence of an XX/XY sexual system in *H. soniae* and the regulatory mechanisms underlying its atypical meiotic behaviour.

## 1 INTRODUCTION

The sucker mouth catfish (Siluriformes, Loricariidae) are the second‐largest Neotropical fish family covering ~1000 species (Roxo et al., 2019). *Hypostomus* La Cépède, 1803, comprises 160 valid species that exhibit high morphological variation and contrasting body colouration (Fricke et al., 2025). Despite taxonomic uncertainty, some authors support the validity of four species super groups within *Hypostomus*: (1) group *Hypostomus cochliodon*, (2) group *Hypostomus hemiurus*, (3) group *Hypostomus aurogutatus* and (4) group *Hypostomus plecostomus* (Cardoso et al., 2012; Lujan et al., 2015; Montoya‐Burgos et al., 2002; Queiroz et al., 2020).

Cytogenetic data are important as a taxonomic marker, and chromosomal evolution may have implications for the speciation process. Due to the high diversification observed in Loricariids, karyotypic studies may contribute to elucidate taxonomic issues in this group. Previously cytogenetic investigations revealed an extensive and complex karyotype evolution among the *Hypostomus* lineages (for revision see, Cereali et al., 2008; Milhomem et al., 2010; Alves, Borba, Oliveira, et al., 2012; Traldi et al., 2013; Brandão et al., 2018; Lorscheider et al., 2018; Oliveira et al., 2019). In this genus the basal diploid number is 2*n* = 64, which is reported in the species *Hypostomus soniae* Hollanda‐Carvalho & Weber, 2004, *Hypostomus faveolus* and *H. cochliodon*, and *Hypostomus* sp. Xingu‐1 to 2*n* = 84 in *Hypostomus perdido* Zawadzki et al. (2014). Differentiated sex chromosome is a rare condition in fish karyotypes (Kirkpatrick et al., 2014); however, in *Hypostomus* a few cases of the chromosome sex system were reported: XX/XY in *Hypostomus* aff. *ancistroides, Hypostumus macrops* (Rocha‐Reis et al., 2018; Michele et al., 1977, respectively) and *H. soniae* (Oliveira et al., 2019) and Z/ZW system in *Hypostomus* sp., *Hypostomus* cf. *plecostumus* and *Hypostomus* aff. *ancistroides* (Artoni et al., 1998, Oliveira et al., 2015; Kamei et al., 2017, respectively).

Differentiation and evolution of sex chromosomes have been studied by analysing meiotic behaviour and synaptonemal complex of sexual pair, which is considered a robust approach for demonstrating chromosomal sex system in fish (Araya‐Jaime et al., 2015).

In these organisms, the identification of sex chromosomes is limited by frequently small chromosome sizes and similar morphology between homologues; however, the occurrences of atypical synapses are good evidence of sex chromosome (Lisachov et al., 2024).

Cytogenetic characterization of blue‐eyed red‐fin pleco *H. soniae* originating from the upper Tapajós River basin revealed a complex heterochromatin polymorphism in pair 26 of the karyotype. This heterochromatic region exhibited distinct sizes between male and female individuals, which was interpreted as evidence for an XX/XY sex system in this species (Oliveira et al., 2019).

*H. soniae* is a popular ornamental pet fish named as blue‐eyed red‐fin pleco or violet pleco, which is numbered as L‐137 for the aquarium market. This species is confined to the Tapajós River basin, with little information on its natural history; the Brazilian government authorizes the sale of this species in the aquarium market (Sousa et al., 2018). The present study demonstrates the karyotypic arrangement and the meiotic stages of *H. soniae* collected from its type locality (de sua localidade tipo) to compare with the Teles‐Pires River population and provide insights into the proposed nascent XX/XY sex system.

## 2 MATERIALS AND METHODS

### 2.1 Sampling

**FIGURE 1:**
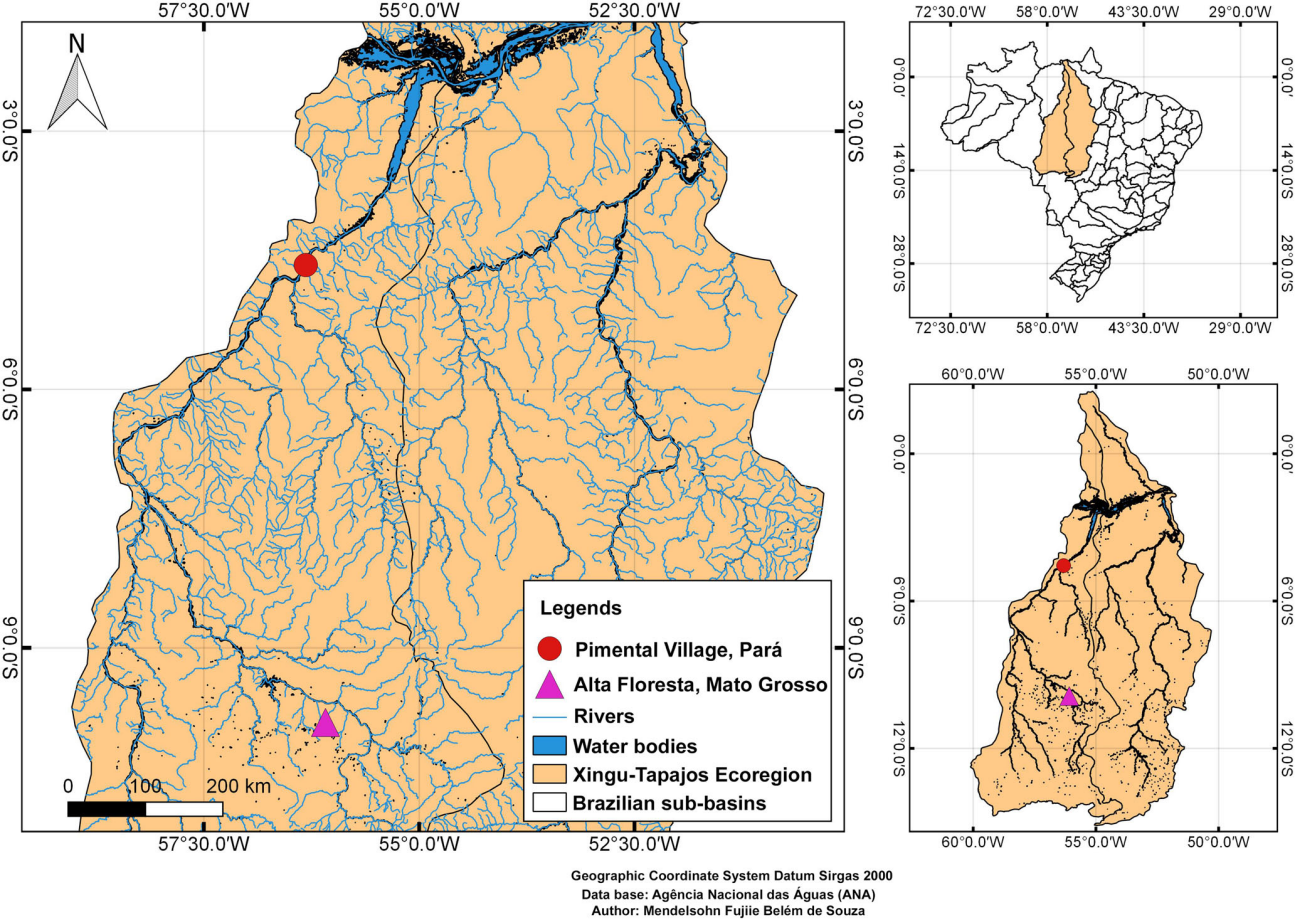
Map of collection site of *Hypostomus soniae* in the Tapajós River analysed in the present study (red circle) and from Teles Pires River previously studied by Oliveira et al. (2019) (pink triangle). The Brazilian territory and Tapajós River basin are delimited in boxes at the upper right corner and down right corner, respectively. This map was assembled using public shapefiles from Agência Nacional das Águas (Brazilian government) and created with free software QGIS.

We analysed 13 *H. soniae* individuals (6 males, 7 females, weight: 83.1 ± 42.9 g, standard length: 134.7 ± 22.7 mm; Table S1). The fishes were collected at Pimental village, in the municipality of Trairão, on the right bank of the Tapajós River, Pará State (4°34′5.9″ S, 56°15′44.4″ W) (Figure 1). Alive specimens were transported to the laboratory at UFOPA‐Campus Tapajós at Santarém and were acclimated in aerated aquaria prior to the chromosome preparation procedures. The specimens were collected under SISBIO authorization licence number 82273‐1. The animals collected were anaesthetized/euthanized using Eugenol immersion (Griffiths, 2000) following ARRIVE guidelines approved by the Ethic Committee of Animal Research‐CEUA‐UFOPA (process no. 1020180043).

### 2.2 Chromosome preparations

We performed mitosis stimulation by subcutaneously injecting yeast *Saccharomyces cerevisiae* (Florax‐HEBRON) in a concentration of 0.01 mL/g for 48 h (Lee & Elder, 1980). Metaphasic chromosomes were obtained from kidney tissue following Gold et al. (1990) with minor adaptations as follows: the kidney cell homogenate was suspended in 8 mL of Hank's solution; then 400 μL of 0.0125% Colchicine (SERVA) was added, and the mixture was incubated at 37°C for 20 min. The cells were subjected to hypotonization with 0.075 M potassium chloride and then fixed. The cell suspension was stored at −20°C for further analysis.

Meiotic cells for synaptonemal complex analysis were prepared following Araya‐Jaime et al. (2015) with minor adaptations. Gonads were dissected using 500 μL of 1× phosphate‐buffered saline (PBS) and stored on ice. The meiotic suspensions were hypotonized with 0.075 M KCl for 30 min at room temperature and then transferred to 300 μL of 100 mM sucrose solution (pH 8.5). The slides were pre‐coated with 2% paraformaldehyde (pH 8.5) and mixed with 30 μL of sucrose cell suspension. The slides were incubated in a humidified chamber for 2 h at room temperature, washed with 0.08% Photo‐flo solution (Kodak) for 5 min and stored at −20°C until immunostaining.

### 2.3 Staining techniques and fluorescent in situ hybridization banding

Conventional staining was performed using 5% Giemsa diluted in phosphate buffer (pH 6.8). Heterochromatic regions were detected using C‐banding with 5% barium hydroxide at 42°C and 2× 0.3 M sodium chloride and 0.003 M trisodium citrate at 60°C and then stained with 5% Giemsa for 20–30 min (Sumner, 1972). The nucleolar organizing regions (NOR) were detected using silver nitrate impregnation following Howell and Black (1980). The guanine‐cytosine/adenine‐thymine (GC/AT)‐rich domains were localized with chromomycin A_3_ (CMA3) and 4′‐6‐diamidino‐2‐phenylindole (DAPI) counterstained with methyl green (Donlon & Magenis, 1983; Schweizer, 1976).

Repetitive DNA domains were chromosomally mapped using fluorescent in situ hybridization (FISH) following Pinkel et al. (1986). FISH probes to recombinant DNA (rDNA, 5S, 18S), telomere and histone (H1, H3) were prepared using polymerase chain reaction (PCR) in a reaction mixture with 12.5 μL of 2× PCR Master Mix (Fermentas), 0.5 μL of each primer (5 μM), 100–200 ng of genomic DNA, 0.25 μL of Taq Polymerase 5 U/μL (Kapa Biosystems) and ultrapure water to constitute 25 μL of the final volume. PCR cycling was programmed as follows: rDNA 5S and 18S (95°C/1 min; 94°C/1 min, 56–57°C/1 min, 72°C/90 s, ×35 cycles; 72°C/5 min) and histones H1 and H3 (95°C/4 min; 95°C/1 min, 55–60°C/1 min,74°C/2 min, ×30 cycles; 74°C/5 min). Detailed information on primer sequence, amplicon size and references for all the repetitive probes is presented in Table S2. Telomere probes were prepared following Ljdo et al. (1991) and Martins and Vicari (2012).

PCR products were inspected using 1% agarose gel electrophoresis, and the positive reactions were performed for probe labelling with biotin‐14‐dATP using nick translation with Bionick Labeling System (Invitrogen) or with digoxigenin‐11‐dUTP using DIG‐Nick Translation Mix (Roche, Mannheim). FISH reactions were performed using Avidin‐Fluorescein conjugate and anti‐digoxigenin‐Cy3 for producing green and red spectra, respectively. Chromosomes were counterstained with DAPI and mounted on a Vectashield H‐1000 (Vector Labs). At least 30 metaphases were analysed per specimen, and the karyotype was arranged following Oliveira et al. (2019). In karyotype analysis, the metaphases with the best chromosome extension were captured in bright field using a MOTICAM 10MP digital camera coupled to a Zeiss Axioskop 40 microscope. Images of FISH reactions with at least five metaphases from each experiment were obtained using a Nikon EclipseCsI epifluorescence microscope coupled to a monochrome CCD camera DS‐Qi1Mc.

Image capture and brightness/contrast adjustments were performed using Nikon NIS‐Elements software. Karyotype assembly/editing was performed using Adobe Photoshop CS6.

### 2.4 Meiotic immuno‐cytogenetic analysis

The blocking stage was performed using 5% bovine serum albumin solution at room temperature for 30 min. Meiotic chromosomes were immunodetected using primary antibodies: rabbit anti‐structural maintenance of chromosomes protein 3 (SMC3) (Abcam, ab9263) at 1:200, rabbit anti‐γH2AX (Abcam, ab2893) and rabbit anti‐H3K9ac (Millipore, 07‐352) at 1:50. Slides were incubated with antibodies for 2 h at 37°C in a humidified chamber. After having been washed in PBS, the slides were incubated with the secondary antibodies (1:100 PBS‐Tween) for 2 h at 37°C. Chromatin was counterstained by incubating with the Vectashield Antifade Mounting Medium containing DAPI. Immuno‐FISH with SMC3 antibody and 18S rDNA probe was performed according to the protocol described by Araya‐Jaime et al. (2015). Immunofluorescence images for meiotic analysis were captured using a Zeiss Imager D2 microscope (Zeiss, Oberkochen, Germany) and an AxioCam 503 camera (Zeiss) and processed using Zen 2.0 software (Zeiss).

## 3 RESULTS

### 3.1 Karyotypic analysis

**FIGURE 2:**
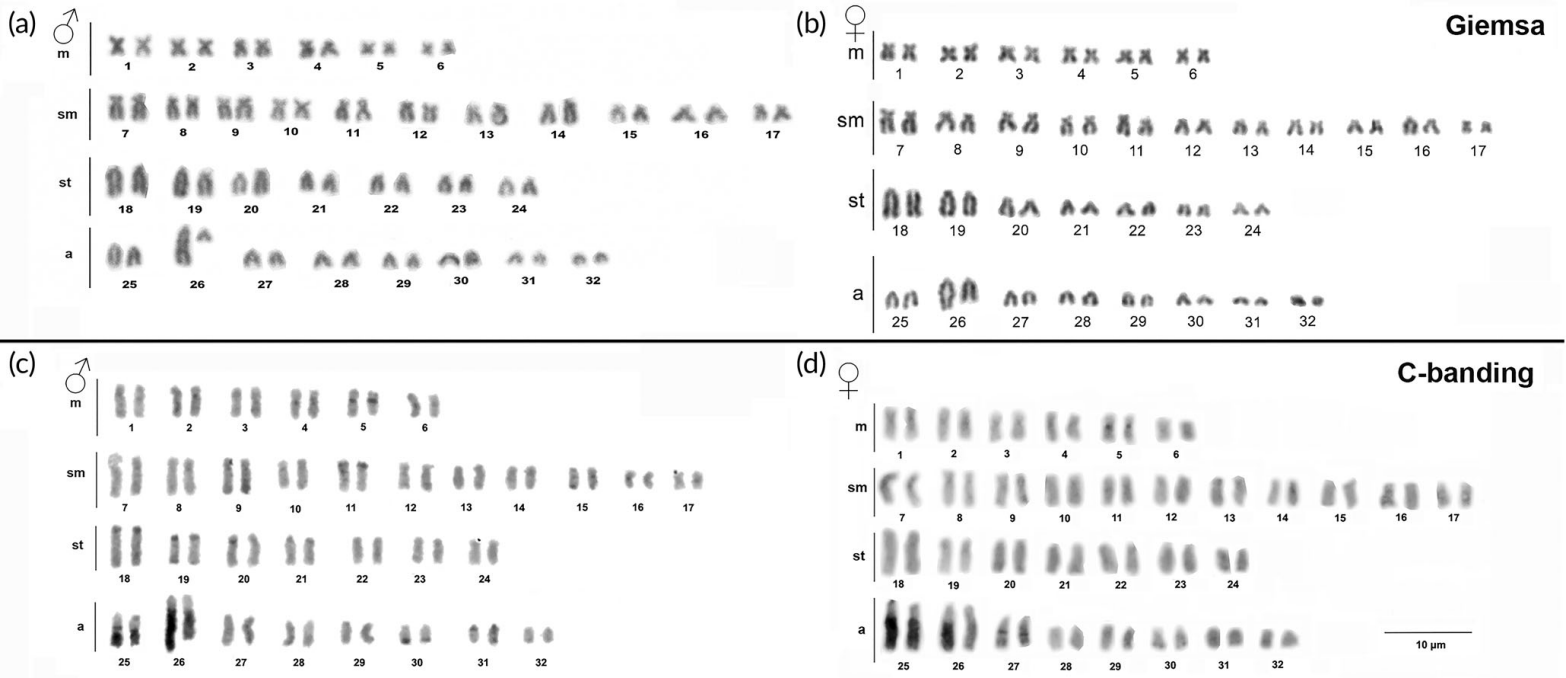
Part labels a‐d, Conventional Giemsa staining (a‐b) and C‐banding on chromosomes of *Hypostomus soniae* from Tapajós River, males and females (c‐d).

*H. soniae* from Tapajós River showed a karyotype with 2*n* = 64, a fundamental number (FN) of 112 and a karyotype arrangement of 12 m + 22sm + 14st + 16a (Figure 2a‐b). Conventional staining revealed conspicuous size heteromorphism in acrocentric pair 26, which was observed in both male and female. C‐banding revealed small amounts of heterochromatic material, which is largely disposed in the pairs 25 and 26; however, tiny C‐bands could be observed in a few pairs at the centromeric (5, 18, 19 and 31) and distal regions (11, 15 and 30) (Figure 2c‐d).

**FIGURE 3:**
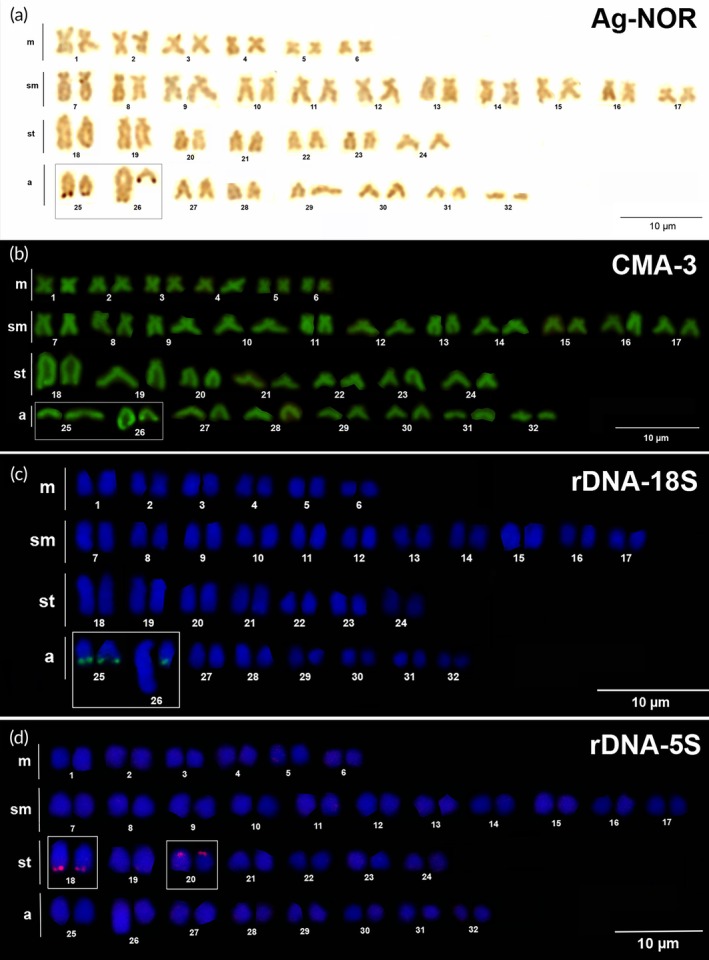
Karyotypes of *Hypostomus soniae* showing (a) nucleolus organizing regions, (b) CMA3 (chromomycin A_3_) sites, (c) FISH (fluorescent in situ hybridization) rDNA (recombinant DNA) 18S and (d) rDNA 5S. Female specimens (a, c, d) and male specimens (b).

The nucleolus organizing regions were detected using Ag‐NOR staining that showed four impregnated sites localized in the terminal region of the long arm in acrocentric pairs 25 and 26 (Figure 3a). Whereas the rDNA cistrons in pair 25 showed active NOR in both homologues, the heteromorphic pair 26 showed a strong mark only in the shortest homologue, and the counterpart, the longest homologue, presented a tiny NOR rarely detected using silver impregnation. CMA3 showed significant marks coinciding with the NOR positions; however, in contrast, in the longest homologue of pair 26 the CMA3 brightness extends from the telomere to half size of the long arm, which is compatible with the heterochromatic C‐band detected in the same position (Figure 3b). Hybridization of rDNA 18S probes confirmed the position of NORs in distal region pairs 25 and 26, which failed to demonstrate rDNA cistrons in the longest homologue of pair 26 (Figure 3c).

**FIGURE 4:**
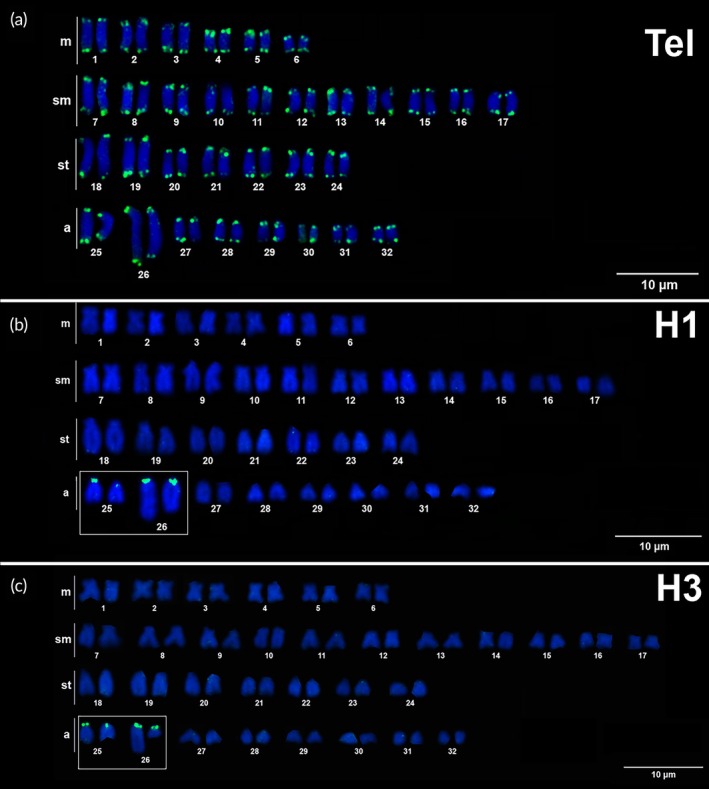
Karyotype of *Hypostomus soniae* (male specimens) hybridized with repetitive DNA probes: (a) telomeric (Tel), (b) histone H1 and (c) histone H3. The hybridization signals (green dots) were detected using Avidin‐FITC and chromosomes stained with DAPI (4′‐6‐diamidino‐2‐phenylindole, blue).

The probes of ribosomal gene 5S were detected in two chromosome pairs, in the distal region in the long arm of pair 18 and the centromeric region of pair 20 (Figure 3d). Telomeres were mapped at just the terminal position of all the chromosome pairs (Figure 4a), whereas histones H1 and H3 were colocalized at similar positions in the centromeric region of chromosomes 25 and 26 (Figure 4b,c).

### 3.2 Immuno‐FISH and meiotic analysis

**FIGURE 5:**
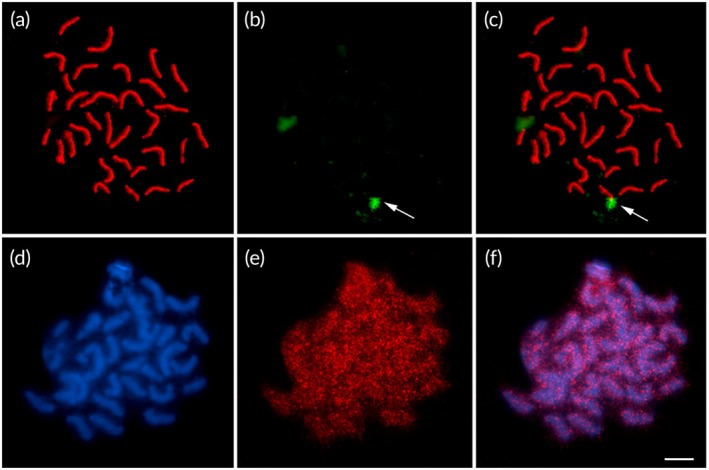
Meiotic analysis in *Hypostomus soniae*. (a) Immunodetection of SMC3 (red), revealing 32 synapsed bivalents: (b) 18S rDNA (recombinant DNA, green), (c) SMC3/18S rDNA immuno‐FISH (fluorescent in situ hybridization), (d) DAPI (4′‐6‐diamidino‐2‐phenylindole)–stained bivalents (blue), (e) H3K9ac (red) and (f) distribution of H3K9ac in meiotic chromosomes of *H. soniae*.

Meiotic analysis using anti‐SMC3 showed that in pachytene cells all 32 bivalents are fully synapsed (Figure 5a). Immuno‐FISH revealed only one pair carrying 18S rDNA (Figure 5b,c). Anti‐H3K9ac antibody showed that this histone modification distributed throughout the entire bivalents during pachytene, with a dispersed and uniform pattern (Figure 5d–f). Signals for γH2AX hybridization on meiotic cells were not detected in this study.

## 4 DISCUSSION

The karyotypic macrostructure of 2*n* = 64 and an FN of112 was conserved in the population from the Tapajós River, which has a karyotype very similar to that found in specimens from Teles Pires River (Oliveira et al., 2019). Such a karyotypic pattern of *H. soniae* is congruent with that of *H. cochliodon* (Paiva et al., 2024). In this regard, karyotypic data support classification of *H. soniae* in the supergroup *H. cochliodon* as evidenced by multilocus phylogeny (Queiroz et al., 2020).

Populations of *H. soniae* from Tapajós and Teles Pires rivers clearly diverged by NOR pattern. The former has NOR consistently labelled as pairs 25 and 26, contrasting with the extensive NOR polymorphism observed in individuals from Teles Pires River, which is characterized by six distinct phenotypes resulting from marks in pairs 14, 15, 25, 26 and 31 (Oliveira et al., 2019).

The most common method to establish NORs in most fish consists of simple systems (Gornung, 2013) and has been considered as a primitive character for Loricariidae (Alves, Borba, et al. 2012). However, multiple NORs are not a rare status and have been detected in many fish groups, for example: Callichthyidae, Characidae, Erythrinidae, Lebiasinidae and Sternopygidae (Almeida‐Toledo & Foresti, 1985; Moreira‐Filho et al., 1984; Galetti et al., 1995; Santos et al., 2016). Multiple NORs had also emerged in the genome of species of the tribe Hypostomini (Rubert et al., 2016). Both NOR patterns (single and multiple) are observed in *Hypostomus*; for example, *Hypostomus strigaticeps* and *Hypostomus nigromaculatus* have a single NOR, whereas *H. ancistroides*, *Hypostomus boulengeri* and *H. cochliodo*n have multiple NORs (Paiva et al., 2024; Pansonato‐Alves et al., 2013).

Intraspecific polymorphism of NOR may occur by variation in number and position of the NORs. The occurrence of NOR polymorphism in *H. soniae* is not an isolated case; such variations had been recorded in *H. nigromaculatus* (Pansonato‐Alves et al., 2013; Rubert et al., 2008) and *H. cochliodon* (Bueno et al., 2014; Rubert et al., 2016). The high mobility of rDNA cistrons in *H. soniae* has been hypothetically explained by the occurrence of transposition events and possibly linked to association with constitutive heterochromatin (Oliveira et al., 2019). The colocalization of rDNA repeats and transposons Tc‐1Mariner associated with heterochromatin supposedly led to the mobilization of rDNA sequence in the genome of *Imparfinis* (Gouveia et al., 2013). Further studies should investigate a possible association between rDNA and transposable elements in the genome of *H. soniae*.

Some species of *Hypostomus* had been mapped for 5S rDNA clusters and revealed two patterns: (1) single 5S‐bearing pair and (2) multiple pairs (Bueno et al., 2014; Ferreira et al., 2019). *H. soniae* exhibited 5S marks in two subtelocentric pairs, allied with *H. cochliodon* in the ‘multiple 5S pattern’, however diverged from this in number and position of 5S clusters (Bueno et al., 2014). The occurrence of 5S at a centromeric region in a metacentric pair is a common feature observed in several species of *Hypostomus* (Bueno et al., 2014; Ferreira et al., 2019); however, it is absent in the karyotype of *H. soniae* and *H. cochliodon*.

The telomeric sequences were detected in the terminal portions of all chromosomes; additionally no interstitial telomeres could be observed, similar to that evidenced for other species such as in *Pseudacanthicus spinosus*, *Pseudacanthicus leopardos* and *Transancistrus santarosensis* (Silva et al., 2022; Tursellino et al., 2023).

The physical mapping of histone H1 and H3 genes in *H. soniae* colocalized in the centromeric region of two pairs of chromosomes, similar to the H3 location mapped in *H. strigaticeps* and *H. nigromaculatus*. The simultaneous occurrence of H1 and H3 has been reported for some specimens of Loricariidae, as most recently in *Spatuloricaria* sp. from the Caripetuba River in Abaetetuba‐PA (Almeida et al., 2023; Pansonato‐Alves et al., 2013).

The chromosomal colocalization of histone gene clusters has exhibited high regularity in the species analysed, being reported as conserved, and functionally H1 and H3 act in the organization of the chromatin structure and in the epigenetic regulation of gene expression, where this becomes possible due to the intrachromosomal spatial proximity that can promote coexpression due to the sharing of promoters and transcription factors (Cabrero et al., 2009; Dai et al., 2014; Nagoda et al., 2005).

Thus, the presence of clusters of H1 and H3 colocalized in chromosome pairs 25 and 26 in *H. soniae* may be attributed to the organization of the large blocks of heterochromatin found in these chromosomes.

We are the first to investigate meiotic chromosome of *H. soniae*, which is assumed to be the initial stage of XX/XY differentiation (Oliveira et al., 2019). The emergence of an XX/XY sex system is linked to chromosomal rearrangements followed by heterochromatinization (Filho, 2017). This process can generate different morphologies of the homologues belonging to the sexual pair, as observed in *H. soniae*. Consequently, the absence of homology in the chromosomal region affected by this heteromorphism can promote recombination suppression and pairing errors between both X and Y (Lisachov et al., 2024). Our results showed that immuno‐FISH failed to demonstrate evidence of an asymmetric synapsis of a sexual pair in pachytene; therefore, we did not find in the present data a clear support of the XX/XY system in *H. soniae*. Indeed, it was observed that the pairing of all bivalents is completed during pachytene, suggesting the occurrence of synaptic adjustment possibly with non‐homologous synapsis and equalization of the lengths of the lateral elements of the components of the putative sexual pair (chromosome 26). This phenomenon was previously observed for heteromorphic XY chromosomes in *Oncorhynchus mykiss* (Oliveira et al., 1995), *Gasterosteus aculeatus* (Nath et al., 2022) and *Nothobranchius furzeri* (Štundlová et al., 2022).

As mentioned earlier, the population of *H. soniae* from the Tapajós River (present study) has three NOR sites co‐located with constitutive heterochromatin at the tips of 25q and 26q, in the latter labelled in just one homologue. The hybridization of 18S probes on pachytenes yielded a single FISH signal, which we interpreted, given the size of this mark, as corresponding to the NORs of pair 25. The lack of detection of the ribosomal cistrons in 26q could be associated with technical limitations of the immuno‐FISH protocol. As noted by Ye et al. (2010), weak signals from DNA probes in immuno‐FISH can result from factors such as inadequate chromosome denaturation or insufficient drying of the slides before immersion in the denaturation solution.

We analysed the epigenetic marks on meiotic chromosomes of *H. soniae*. In mammals, amphibians and birds, it is observed that during some subphases of prophase I, the asynaptic chromatin of the XY body does not present transcriptional activity (Noronha et al., 2020; Turner, 2015). This silencing of gene expression is mediated by γH2AX, which attracts repair proteins such as BRCA1, inducing the formation of epigenetic modifications related to the inactivation of gene expression, such as H3K9me3. In the present study, anti‐γH2AX antibodies revealed no positive signals in *H. soniae* meiocytes. A similar result was observed in lampreys (Matveevsky et al., 2023). Thus, it is possible that silencing mechanisms in *H. soniae* can be distinct from those reported for mammals and birds. In contrast, anti‐H3K9ac showed that this marker is widely distributed throughout the bivalents. This finding suggests that gene transcription is active during the early prophase I, but which is stopped in the later stages of meiosis in all the bivalents, as observed in other Loricariidae (Almeida et al., 2023). Other markers of active chromatin transcriptional activity showed similar results during gametogenesis of *Characidium gomesi* (Serrano et al., 2016) and lamprey (Matveevsky et al., 2023).

The information cited does not confirm the XX/XY sexual system in *H. soniae*, but it may be a system that is in the early stages of formation.

## 5 CONCLUSIONS

*H. soniae* from the Tapajós and Teles Pires rivers share conservative karyotypic macrostructure with similar diploid number and karyotypic formula; however, there are divergences in number and position of NORs or 18S rDNA clusters. Both populations have conspicuous heterochromatic blocks in the acrocentric pair 26, which exhibit high polymorphism of size variation that may be interpreted as indicative of an XX/XY system. However, this hypothesis is not corroborated by the synaptic behaviour of the putative sexual pair on pachytene meiocytes, because all the bivalents are fully synapsed and transcriptionally active. Further cytogenetic and molecular investigations are necessary to clarify the proposed emergence of an XX/XY sexual system in *H. soniae* and the regulatory mechanisms underlying its atypical meiotic behaviour.

## AUTHOR CONTRIBUTIONS

Luan Aércio Melo Maciel: Aquisição, análise ou interpretação dos dados do trabalho, Aprovação final da versão a ser publicada e concordância em ser responsável por todos os aspectos do trabalho. Renata Coelho Rodrigues Noronha: Contribuições substanciais para a concepção ou delineamento do estudo. Bruno Rafael Ribeiro de Almeida: Aquisição, análise ou interpretação dos dados do trabalho. Manoella Gemaque Cavalcante: Aquisição, análise ou interpretação dos dados do trabalho. Cleusa Yoshiko Nagamachi: Elaboração de versões preliminares do artigo ou revisão crítica de importante conteúdo intelectual. Julio Cesar Pieczarka: Elaboração de versões preliminares do artigo ou revisão crítica de importante conteúdo intelectual. Luís Reginaldo Ribeiro Rodrigues: Contribuições substanciais para a concepção ou delineamento do estudo, elaboração de versões preliminares do artigo ou revisão crítica de importante conteúdo intelectual.

## FUNDING INFORMATION

The authors are grateful to PhD. Roberta B. Sciurano (University of Buenos Aires, Argentina) and PhD. Cesar Martins (State University of São Paulo) that kindly donated the antibodies utilized. Some specimens were kindly provided by Tapajós Aquariuns Company (Santarém‐PA) and Projeto Arapaima (Belém‐PA). LAMM received a doctoral fellowship from Conselho Nacional de Desenvolvimento daPesquisa (CNPq). LRRR was funded by Coordenação de Apoio de Pessoal deNível Superior (CAPES) – Programa Pró‐Amazônia (Auxpe 3318/2013). CYN (307170/2021‐7) and JCP (307154/2021‐1) are grateful to CNPq for Productivity Grants.

## Supporting information


**Table S1.** Morphometric data regarding the field code, sex, standard length and weight of the *Hypostomus soniae* specimens analysed in the present study.

**Table S2.** Primers of repetitive DNA markers used in PCR (polymerase chain reaction) for FISH (fluorescent in situ hybridization) experiments.
