# Supplementary material for: New insights into the putative XX/XY sex chromosomal system in blue‐eyed red‐fin pleco Hypostomus soniae (Siluriformes, Loricariidae)
Source: J Fish Biol. 2025 Sep 13;108(1):92–102. doi: 10.1111/jfb.70226 (PMC13033946; doi:10.1111/jfb.70226)
Supplement: Supplementary file 1 — Table S1. Morphometric data regarding the field code, sex, standard length and weight of the Hypostomus soniae specimens analysed in the present study. [file JFB-108-92-s002.docx]

(Suppl file - 1).

Suppl Tab 1. Morphometric data regarding the field code, sex, standard length and weight of the *Hypostomus soniae* specimens analyzed in the present study.

| **Sample ID** | **Sex** | **SL (mm)** | **Weigth (g)** |
| --- | --- | --- | --- |
| PMT-138 | F | 170 | 180 |
| PMT-139 | F | 140 | 80 |
| PMT-140 | F | 110 | 42 |
| PMT-141 | M | 160 | 115 |
| PMT-142 | M | 150 | 89 |
| PMT 143 | F | 135 | 79 |
| ARAP 1 | M | 140 | 68 |
| ARAP-3 | F | 130 | 48 |
| ARAP-4 | F | 130 | 59 |
| ARAP-5 | M | 130 | 56 |
| PMT 147 | M | 165 | 130 |
| PMT 149 | F | 160 | 132 |
| PMT-36 | M | 92 | ND |
