# Supplementary material for: New insights into the putative XX/XY sex chromosomal system in blue‐eyed red‐fin pleco Hypostomus soniae (Siluriformes, Loricariidae)
Source: J Fish Biol. 2025 Sep 13;108(1):92–102. doi: 10.1111/jfb.70226 (PMC13033946; doi:10.1111/jfb.70226)
Supplement: Supplementary file 2 — Table S2. Primers of repetitive DNA markers used in PCR (polymerase chain reaction) for FISH (fluorescent in situ hybridization) experiments. [file JFB-108-92-s001.docx]

**Supplemental file 2:**

Primers of repetitive DNA markers used in PCR for FISH experiments.

| **Primer** | **Sequence** | **Amplicon size (pb)** | **Reference** |
| --- | --- | --- | --- |
| Histone H1 | 5’ -GAGTTGGATCGGGCTCAGGAGCG-3’  5’ -CGGTCAGCCTCTTCAGCAATGTGCTT-3’ | 620 | Hashimoto *et al*. (2011) |
| Histone H3 | 5′-ATG GCT CGT ACC AAG CAG AC(ACG) GC-3′  5′-ATA TCC TT(AG) GGC AT(AG) AT(AG) GTG AC-3′ | 376 | Cabral-de-Mello *et al*. (2010) |
| 5S rDNA | 5′-GCC ACA CCA CCC TGA ACA C-3′  5′-GCC TAC GAC ACC TGG TAT TC-3′ | 120 | Suarez *et al.* (2017) |
| 18S rDNA | 5’CCG CTT TGG TGA CTC TTG AT 3’  5’CCG AGG ACC TCA CTA AAC CA 3’ | 1400 | Martins e Vicari (2012) |
| Telomere | F (5’TTAGGG3’)5  R (5’CCCTAA3’)5 | nd | Ljdo *et al*., (1991) |

nd (non determined)
